# Direct Observation of Deformation in Microgel Filtration

**DOI:** 10.1038/s41598-019-55516-w

**Published:** 2019-12-12

**Authors:** John Linkhorst, Jonas Rabe, Lukas T. Hirschwald, Alexander J. C. Kuehne, Matthias Wessling

**Affiliations:** 1 RWTH Aachen University, Chemical Process Engineering, Aachen, 52074 Germany; 2 DWI – Leibniz Institute for Interactive Materials, Aachen, 52074 Germany; 3 Ulm University, OC3 – Institute of Organic and Macromolecular Chemistry, Ulm, 89081 Germany

**Keywords:** Chemical engineering, Gels and hydrogels

## Abstract

Colloidal filtration processes using porous membranes suffer from productivity loss due to colloidal matter retention and continuous build-up by the retained matter. Especially during filtration of soft matter, the deformation of the individual colloids that make up the filter cake may be significant; however, this deformation and its impact remain unresolved so far. Yet, understanding the deformation on the single colloid level as well as on the ensemble level is important to be able to deconvolute filter cake properties from resistance increase of the membrane either by simultaneous internal adsorption or blocking of pores. Here, we report on the compression of a filter cake by filtrating soft microgels in a microfluidic channel in front of a model membrane. To study the single colloid deformation amorphous and crystalline domains were built up in front of the membrane and visualized on-line using confocal fluorescence microscopy while adjusting the degree of permeation, i.e., the transmembrane flux. Results show locally pronounced asymmetric deformation in amorphous domains, while the microgels in colloidal crystals approached regular polyeder shape. Increasing the flux beyond the maximum colloid deformation results in non-isochoric microgel behavior. The presented methodology enables a realistic description of complex colloidal matter deposits during filtration.

## Introduction

Membrane filtration is a rapidly growing and powerful technology with important applications ranging from water purification, disinfection, beverage clarification to healthcare technologies, for example, hemodialysis. During filtration, colloids and particulates dispersed in the feed are retained and concentrated in front of the membrane, while the permeate phase becomes purified. The concentration of the dispersed colloids and solids causes a filter cake to build up on the membrane surface. Progressively, the filter cake reduces the filtration performance over the course of the process. Depending on the specific separation task, the filter cake contains hard or soft matter, such as colloids or matter of biological origin, for example, cells, extracellular polysaccharides, or proteins. In contrast to hard particulates, the soft matter compacts on the membrane due to the applied transmembrane pressure and the permeation related pressure loss, which increases the hydraulic resistance and reduces the filtration productivity. Additionally, this soft matter compaction is suspected of inducing irreversible fouling with colloidal aggregates interlocking with the membrane porosity, which cannot be removed from the membrane surface during backwashing cycles^1^. Such occurrences are hypothesized to form the origin of long-term degradation processes of the membrane, which ultimately necessitates its replacement^2^. Comprehending these colloidal filtration processes and in particular, confirming the above described microscopic events (which are currently only described using intricate simulations) is of utmost importance to support today’s evidence-based filtration models with detailed microscopic understanding.

To study the behavior of such real-world soft filter cakes with their complex composition, synthetic soft colloids with previously established properties have been used as model foulants in several experimental and theoretical simulation studies^3–9^. Depending on their size, these synthetic soft colloids represent ideal entities to mimic the mechanical and hydraulic properties of soft biological matter, ranging from proteins to cells and biofilms. Recently, several groups reported on the deformation of soft colloids, using microgels, which are swollen polymer networks with dimensions on the micrometer and sub-micrometer scale. In a mixture of small microgels with a few larger microgels, the latter is compressed without deformation^10^. Faceting has been observed in suspensions of microgels with the same size and mechanical properties in random close packings under osmotic pressure^11^. Whether the onset of isotropic compression occurs before faceting^12^ or after^11^ is still under debate and has not been resolved to date. Deformation behavior of hollow microgels has been studied in overcrowded environments by light scattering in combination with molecular modeling, adding an additional means to study mechanical properties of microgels^13,14^. On the application side, Hinge *et al*. reported on the filtration resistance of such soft colloidal filter cakes^15^. They analyzed pressure data and imaged the filter cake *ex-situ* using electron microscopy. Our own previous study indicates that microgel filter cakes can be partly crystalline, depending on the filtration conditions^16^.

To understand filter-cake build-up and compression, online imaging of individual colloids and their shape in crystalline and amorphous cake morphology is of great importance. The shape of the colloids and the morphology of the cake strongly influence permeability and pore size in the cake. With in-depth knowledge of this, it will be possible to avert high hydraulic resistance and tortuosity of filter cakes in new membranes of the future. However, to date, there are only a few recent online studies available that monitor membrane filter cake generation and compaction over time^17–19^. More knowledge in this field would give crucial information on how to design membranes of the future that avoid degenerative fouling conditions. Here, we report on spatially resolving the compression of a colloidal filter cake by rejecting soft microgels in a microfluidic channel in front of a pre-clogged model membrane. The model membrane is represented by a set of parallel pores in a microfluidic channel, while the microgels mimic biological or natural soft matter. The focus lies on colloid-colloid interactions in amorphous and crystalline regions of the filter cake, as well as the bulk behavior of microgels.

## Results and Discussion

The colloidal model filter cake is produced by flowing the soft microgels through a microchannel towards the model membrane on a microfluidic chip. The membrane pores on the chip have a narrowest point of 11 μum. These pores are then obstructed by flowing in ~20 μm polystyrene particles. The build-up of the colloidal filter cake against these obstructions is visualized using confocal laser scanning microscopy (see Methods Section).

The morphology of the filter cake can be both amorphous and crystalline, depending on the flow conditions during filtration. In general, high filtration fluxes with high colloid loading induce amorphous filter cake formation, whereas low filtration fluxes yield more ordered or crystalline filter cakes^16^. This work first focusses on single colloid deformation in amorphous domains, followed by studying their deformation in crystalline domains. Subsequently, the deformation of the filter cake is studied as a whole, which is generally considered as its bulk compression.

### Colloid deformation in amorphous domains

To investigate the deformation of individual microgels, experiments were conducted using core-shell microgels with a hard fluorinated polyacrylate core of 500 nm incorporating a green fluorescent dye (FITC) and a polyNIPAM shell yielding soft microgels of ~2 μm. 50% of these microgels have been labeled in the shell with a reactive green or red fluorescent dye to be able to visualize these microgels using confocal microscopy.

**Figure 1:**
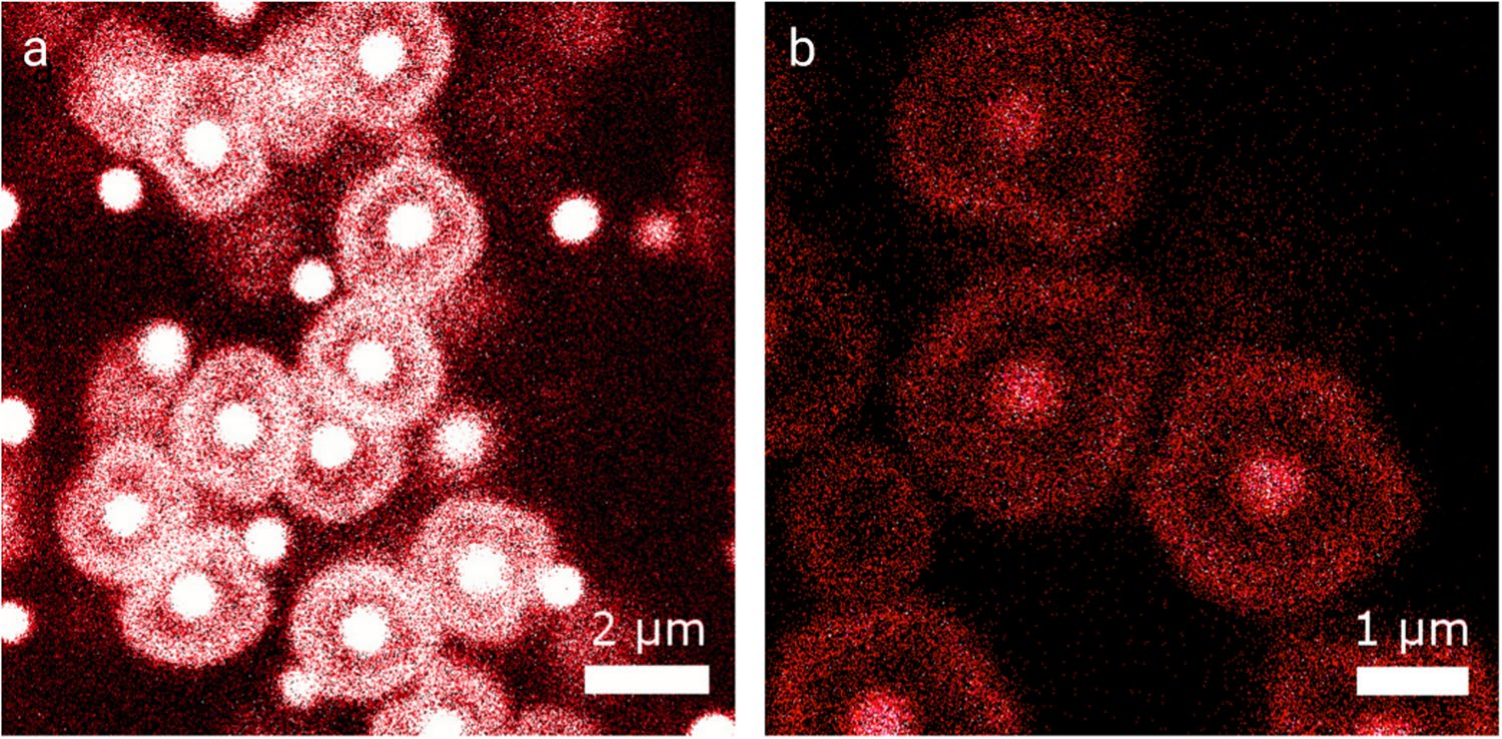
Two deformed amorphous regions in the filter cake with FITC shell labeled microgels. (**a**) Δ*p* = 225 mbar (**b**) Δ*p* = 270 mbar.

When filtering these microgels against the microfluidic membrane, the microgel mixture arranges into a randomly packed dense colloidal filter cake, as shown for two similar compression pressures of Δ*p* = 225 mbar and Δ*p* = 270 mbar in Fig. 1. The labeling procedure with FITC yields microgels with a strong signal at the perimeter of the microgel shell, allowing to investigate the precise deformation of these labeled microgels. Upon compression, the deformation of the soft microgel shell in such randomly close-packed assemblies is inhomogeneous. Due to the irregular distribution of contact points, high local forces lead to strong anisometric deformations of individual colloids, as for example shown in the upper left corner of Fig. 1a. At higher magnification (Fig. 1b) a dark gap between the shells becomes apparent, indicating that the compression energy is stored in deformation of the polymer network constituting the microgel shell rather than leading to interpenetration of the loosely cross-linked polymer networks.

### Colloid deformation in crystalline domains

**Figure 2:**
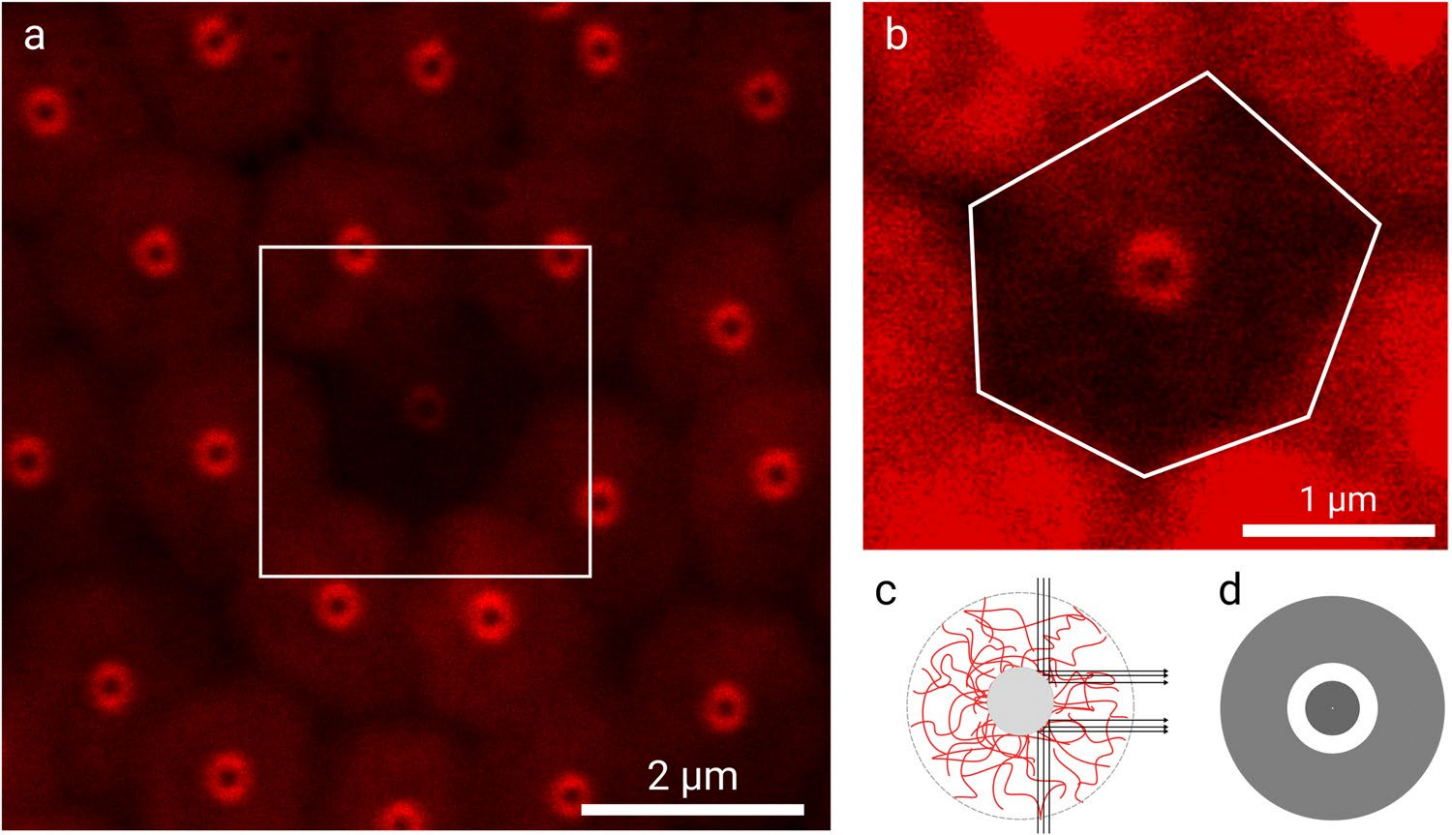
(**a**) Deformation of a crystalline domain at Δ*p* = 90 mbar. All colloids but the one in the center are labeled at the shell with Alexa Flour 647 hydrazide. (**b**) Increased contrast magnification of the center microgel with the perimeter highlighted as a distorted hexagon. (**c**) Graphical representation of the light path coming from adjacent colloids, leading to the donut. (**d**) Ray tracing rendering of a sphere-in-sphere of the situation.

When the colloidal filter cake is produced at significantly lower fluxes and pressures, more ordered filter cakes are observed with hexagonal microgel arrangements, as shown in Fig. 2. Here, 95% of microgels with fluorescently labeled shells (using red fluorescent hydrazide functionalized Alexa Fluor 647 as a dye) and 5% of unlabeled microgels were applied to allow the observation of deformation. A red fluorescent dye of the microgel shell was chosen to visualize the microgel independently of the green fluorescent solid cores. Using this technique, the filter cake build-up was observed by exciting only the dye in the core, preventing photobleaching in the shell.

Interestingly, the green fluorescent cores of the microgels emit a donut-like signal even though they should not contribute to red fluorescence. One possible explanation for this phenomenon is that the red fluorescence of the Alexa Fluor 647 is reflected off the cores and guided into the objective. To confirm this hypothesis, ray-tracing modeling of a small core sphere surrounded by a large shell sphere with the same size ratio as for our microgels was performed. The ray-tracing experiment leads to a bright donut reflection in accordance with what was observed in confocal microscopy experiments, cf. Figure 2a–d. This effect is not visible in Fig. 1 because the core microgels fluoresce at a similar wavelength as the FITC labeled shell.

Inspection of the fluorescent microgel shell permits to study the mechanics and hydraulics of compaction. When constantly increasing the pressure, significant microgel deformation is witnessed, starting at 90 mbar. The deformation can be recorded especially well at the interfaces with microgels that carry no fluorescent dye in the shell. In contrast to the randomly packed state, compaction of the colloidal filter cake leads to a geometrical deformation of the ordered microgels approaching a hexagonal arrangement in a two-dimensional confocal slice, as shown in Fig. 2a,b. Translating this hexagonal deformation into a three-dimensional arrangement, the spherical microgel will be deformed to the shape of either an irregular dodecahedron or a rhombic dodecahedron.

### Bulk compression

The results discussed so far show that on the individual microgel level, microgels in an amorphous colloidal filter cake deform anisometrically while they deform more isotropically in the ordered colloidal filter cake. In the next step, the compression of the bulk filter cake is investigated, which is achieved by building up and compressing the colloidal filter cake either by increasing the pressure step-by-step or successive increasing the flow rate. For each step, the mean intercolloidal distance is determined by image analysis of confocal microscopy scans at the respective pressure and flow conditions. The distance is initially influenced by convection during the build-up, and with increasing pressure and flux, deformation becomes the predominant effect for distance reduction. The morphology of the cake is amorphous for the most part with smaller crystalline domains.

**Figure 3:**
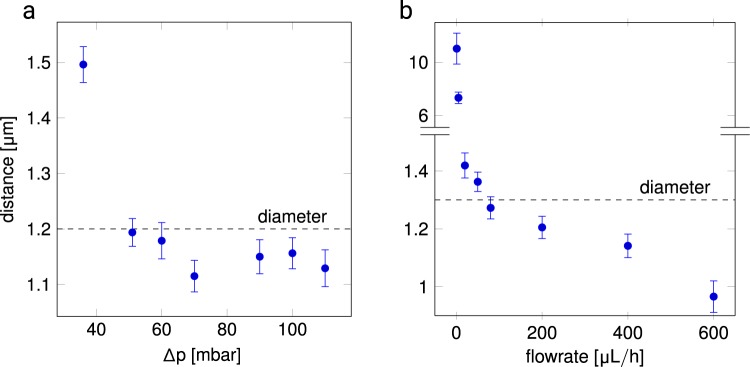
Compression of the bulk colloidal filter cake by (**a**) pressure (microgel diameter 1.2 μm) and (**b**) flow (microgel diameter 1.3 μm). The uncompressed microgel diameter is denoted a dashed line. It is safe to assume that below this line, deformation is the predominant effect. Error bars show standard error with N = 8.

At 50 mbar as well as at 65 μL/h the microgels are in contact with each other, represented by the fact that the distance between the center of two adjacent microgels reaches their individual diameter, see Fig. 3, the dotted lines are a guide to the eye to indicate when the microgels touch. With increasing pressure and flow, the microgels deform, and the colloidal filter cake is progressively deformed. For the increasing pressure experiment fluctuation in the mean inter-microgel distance between 70 mbar and 100 mbar can be observed, see Fig. 3a. This effect may be due to the rearrangement of microgels and the healing of defects to a more ordered colloidal assembly. The maximum pressure that could be reached in this experiment is 110 mbar, beyond which the colloidal filter cake disintegrates and is flushed out of the chip, cf. Fig. 3a. This effect is due to the microfluidic chip design, where the membrane pore size is more than ten times larger than the microgel diameter. This limits the experimental range of pressure and flow rate. With constant flow conditions, a more gradual compaction of the colloidal filter cake is witnessed, see Fig. 3b. The colloidal filter cake is stable up to 600 μL/h, and the distance between the centers of the microgels is reduced by 25.7% with respect to the nominal microgel diameter. If the colloidal filter cake is completely crystalline with a colloidal packing factor of 0.74, the remaining void fraction at this flow rate would be as low as 0.3% if the original volume would be retained (isochoricity).

**Figure 4:**
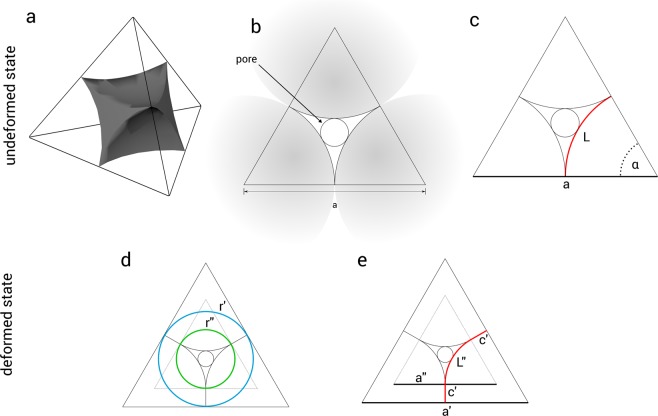
Schematic representations of (**a**) the pore shape in a colloidal crystal, (**b**) the pore diameter for colloidal crystals, (**c**) the pore in the undeformed state with *a* being the distance between two microgel centers and (**b**) and (**c**) in the deformed state, where *a*′ is the center-to-center distance of two deformed microgels.

**Figure 5:**
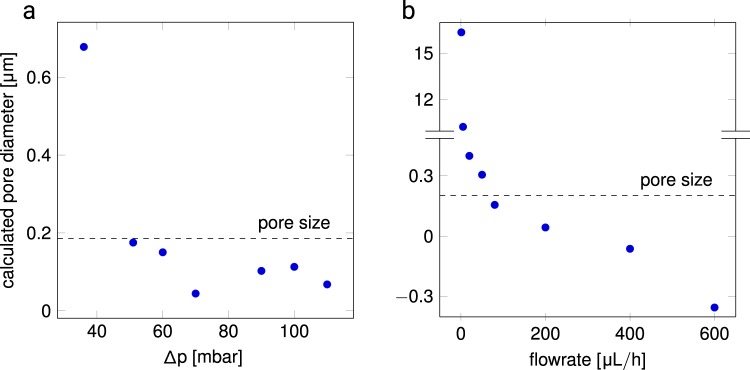
Calculation of mean pore size according to Eq. 1 of (**a**) pressure and (**b**) flow rate data.

It can be hypothesized that liquid flow is much faster through the voids of the compacted microgels compared to the transport through the microgel network. Using the deformation dependent pore diameter, derived from geometrical theory (see Fig. 4) and Eq. 1 it is now possible to plot the pressure and flow rate against the mean pore size of the microgel, as shown in Fig. 5. Here, the pore size of colloidal filter cakes are indicated for non-compressed colloids at 1.2 μm and 1.3 μm (where microgels only touch), see Fig. 5a,b, respectively.

1 $${r}_{pore}=(\frac{\sqrt{3}}{3}-\frac{1}{2})\cdot \frac{\frac{1}{6}\cdot \pi \cdot a-\frac{\sqrt{3}}{3}\cdot a^{\prime} }{\frac{1}{6}\cdot \pi -\frac{\sqrt{3}}{3}}$$

This means, data plotted below this reference line represent compressed colloidal filter cakes.

While the pore diameter remains above 0 for the pressure-driven experiment, it is remarkable that the pore diameter becomes nominally smaller than 0 for the experiment where the flow rate is stepwise increased, as shown in Fig. 5b for flow rates of 400 μL/h and 600 μL/h. This result could be explained by colloids becoming so deformed, that the boundaries of the microgels start to interpenetrate or the deformation is no longer isochoric. As derived from the deformation study of individual microgels, the interpenetration of the polymer networks seems to be energetically unfavorable (see Figs. 1 and 2), therefore non-isochoric deformation is supposed, where microgels shrink, and water is expelled from the network to happen at these high flow rates. This effect would render the pore size calculation incorrect for higher compression rates.

## Summary and Outlook

In this work, experiments on amorphous and crystalline colloidal filter cake compression have been conducted, and equations for calculation of ideal crystalline colloidal filter cake pore sizes have been derived. The compression has been evaluated on single microgel level with the help of labe9led microgel shells and on the bulk filter cake by measuring microgel distances via fluorescent cores. It has been found that compression is supposedly non-isochoric at higher flow rates in the chip.

These results are of great importance for understanding the filtration of soft colloidal matter through membranes since the knowledge about the consequence of compressibility of colloidal filter cakes is limited at the single microgel level as well as the resulting change in hydraulic resistance and the filtration cut-off in membrane processes.

Changes in the colloidal filter cake influence overall filtration performance significantly. Additionally, with increasing compression, the shrinking pore diameter adds a pressure or flux dependent size-exclusion layer to the membrane, effectively influencing the cut-off of the membrane.

Further research is required to correct the pore size calculation for non-isochoric deformation and also account for transport though the deformed soft colloids. While the elastic moduli of m-sized soft microgels are difficult to measure and prone to error^20–23^, our new method using confocal image stacks of consecutive compression steps of shell-labeled microgels could enable an *in situ* determination of these parameters.

## Methods

All experiments were conducted in microfluidic chips. In the center of the chip design, an idealized membrane with straight pores was implemented. The cross-section of a single pore was 11 μm by 20 μm. Channel width and height are 583 μm and 20 μm, respectively, resulting in a total cross-sectional area for the flow of 0.01166 mm^2^.

**Figure 6:**
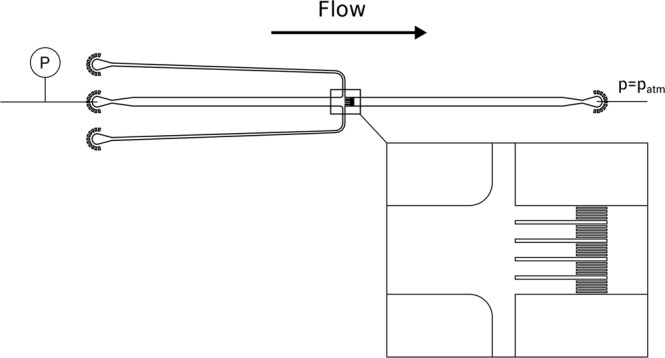
Design of the microfluidic chip for deformation experiments with crossflow channels. The idealized membrane with four protruding obstacles is shown in the magnification.

Figure 6 shows the design of the chip for observation of colloidal filter cake deformation. Core features are the two side-channels that allow flushing of microgels directly in front of the model membrane for faster cake build-up, and four protruding obstacles, effectively dividing the colloidal filter cake into five parts to create five individual filter cakes. The distance of the protruding obstacles was chosen according to the view field of the microscope (Leica SP8 Confocal Laser Scanning Microscope) and the 63x lens.

**Figure 7:**
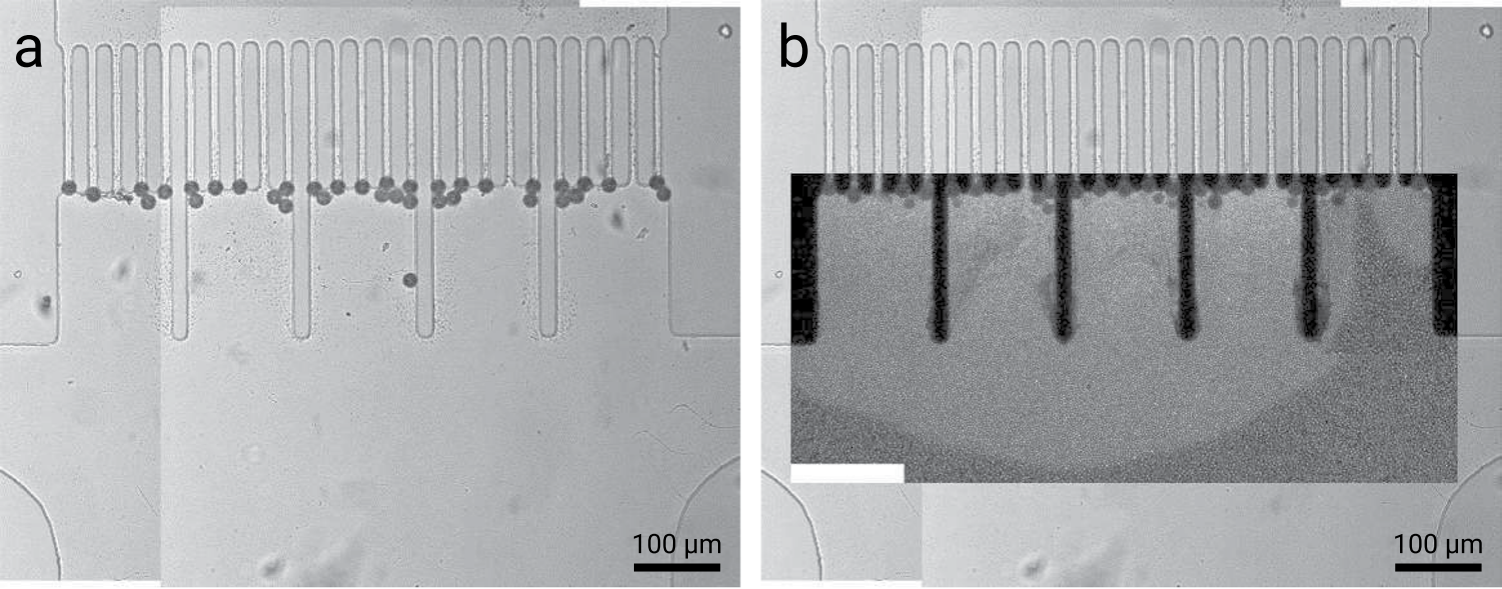
Panorama image collages of an overpressure situation in the filtration device, showing (**a**) bright-field image of the filtration, exposing the polystyrene pre-clogs, and (**b**) inline fluorescence image revealing the cake. The light part of the fluorescence image shows the stationary filter cake; the dark gray part shows water with resuspended microgels. In the rightmost subsection of the cake, a pore opened due to overpressure.

The microfluidic chips were prepared with a standard lithography process and plasma bonded to a 175 μm cover glass. After preparation, the chips were wetted with isopropanol and subsequently flushed with water. Experiments were conducted in two sequential steps, as the smallest features achievable with the masks were 11 μm (25.000 dpi laser photo plot, KOENEN GmbH, Germany). In a first step, pre-clogging was performed with 19.98 μm polystyrene particles (BS-Partikel GmbH, Germany) were flushed into the channel using a 55:45 ratio of water and deuterium oxide (Sigma-Aldrich) for density matching to reduce sedimentation velocity. Once the polystyrene particles block the pores, excess particles were removed through the side channels (see Fig. 7a). The polystyrene particles fulfill the two functions of reducing the pore size and introducing 3D tortuosity to stabilize the filter cake at higher filtration pressures.

In a second step, the actual colloidal filter cake is built up with the microgel dispersion shown in Fig. 7b. For illustration purposes, the fluorescence image, which shows the bright colloidal filter cake, is embedded into the brightfield images of the microfluidic chip. Microgel concentration was 1 mg/mL. The filter cake was built to a height of ~300 μm. The analysis was done under pure water filtration conditions.

To be able to visualize the microgels under the confocal microscope, we prepared polyNIPAM microgels (NIPAM, BIS, Acrylic acid) with a solid green fluorescent polyacrylate core in accordance with a previously reported procedure^24,25^. The polyNIPAM shell is a loosely crosslinked polymer network, which is highly swollen by the medium (water) the microgels are dispersed/dissolved in. This architecture and the chemical functionality of the microgel shell also allow to label the soft polyNIPAM microgel shell with fluorescent dyes using simple isocyanate or hydrazide coupling chemistry. The solid fluorescent polyacrylate cores have diameters of ~350 nm so that their contribution to the mechanical properties of the microgel shell with ~2 μm is negligible.

To visualize deformation at single microgel level, the shells of half of the microgels are labeled with a second dye, which has a different excitation wavelength than the microgel core dye. For experiments on microgel deformation in the amorphous, fluorescein isothiocyanate (FITC, Sigma-Aldrich) was used. For observing deformation in crystalline domains, the more stable Alexa Fluor 647 (ThermoFisher Scientific) was used, as the total experiment time was longer. Photobleaching of both dyes was significant when exposed to laser light. FITC only allowed for single exposure, whereas Alexa Fluor 647 offered an observation time of up to a few minutes.

## References

[1] Mourouzidis-Mourouzis SA, Karabelas AJ (2006). Whey protein fouling of microfiltration ceramic membranes—Pressure effects. Journal of Membrane Science.

[2] Baker, R. W. *Membrane Technology and Applications* (ed. Baker, R. W.) 89-189 (John Wiley & Sons, 2004).

[3] Wyss HM, Franke T, Mele E, Weitz DA (2010). Capillary micromechanics: Measuring the elasticity of microscopic soft objects. Soft Matter.

[4] Roa R, Zholkovskiy EK, Nägele G (2015). Ultrafiltration modeling of non-ionic microgels. Soft Matter.

[5] Dressaire E, Sauret A (2016). Clogging of microfluidic systems. Soft Matter.

[6] Nir O, Trieu T, Bannwarth S, Wessling M (2016). Microfiltration of deformable microgels. Soft Matter.

[7] Bouhid de Aguiar I, Schroën K, Meireles M, Bouchoux A (2018). Compressive resistance of granular-scale microgels: From loose to dense packing. Colloids and Surfaces A: Physicochemical and Engineering Aspects.

[8] Lohaus J, Perez YM, Wessling M (2018). What are the microscopic events of colloidal membrane fouling?. Journal of Membrane Science.

[9] Dersoir B, Schofield AB, Robert de Saint Vincent M, Tabuteau H (2019). Dynamics of pore fouling by colloidal particles at the particle level. Journal of Membrane Science.

[10] Iyer ASJ, Lyon LA (2009). Self-Healing Colloidal Crystals. Angewandte Chemie International Edition.

[11] de Aguiar IB (2017). Deswelling and deformation of microgels in concentrated packings. Scientific Reports.

[12] Conley GM, Aebischer P, Nöjd S, Schurtenberger P, Scheffold F (2017). Jamming and overpacking fuzzy microgels: Deformation, interpenetration, and compression. Science Advances.

[13] Scotti A (2018). Hollow microgels squeezed in overcrowded environments. The Journal of Chemical Physics.

[14] Scotti A (2019). Deswelling of Microgels in Crowded Suspensions Depends on Cross-Link Density and Architecture. Macromolecules.

[15] Hinge M, Christensen ML (2017). Non-ionic soft materials influence on filtration resistance and cake dry matter content. AIChE Journal.

[16] Linkhorst J, Beckmann T, Go D, Kuehne AJC, Wessling M (2016). Microfluidic colloid filtration. Scientific Reports.

[17] de Aguiar IB, Meireles M, Bouchoux A, Schroën K (2019). Microfluidic model systems used to emulate processes occurring during soft particle filtration. Scientific Reports.

[18] de Aguiar IB, Meireles M, Bouchoux A, Schroën K (2019). Conformational changes influence clogging behavior of micrometer-sized microgels in idealized multiple constrictions. Scientific Reports.

[19] Hong X, Kohne M, Morrell M, Wang H, Weeks ER (2017). Clogging of soft particles in two-dimensional hoppers. Physical Review E.

[20] Tominaga T (2008). Effect of substrate adhesion and hydrophobicity on hydrogel friction. Soft Matter.

[21] Hashmi SM, Dufresne ER (2009). Mechanical properties of individual microgel particles through the deswelling transition. Soft Matter.

[22] Guo M, Wyss HM (2011). Micromechanics of Soft Particles. Macromolecular Materials and Engineering.

[23] Abate AR, Han L, Jin L, Suo Z, Weitz DA (2012). Measuring the elastic modulus of microgels using microdrops. Soft Matter.

[24] Go D, Kodger TE, Sprakel J, Kuehne AJC (2014). Programmable co-assembly of oppositely charged microgels. Soft Matter.

[25] Go D (2017). Programmable Phase Transitions in a Photonic Microgel System: Linking Soft Interactions to a Temporal pH Gradient. Langmuir.

